# Probiotic colonization dynamics after oral consumption of VSL#3^®^ by antibiotic-treated mice

**DOI:** 10.20517/mrr.2022.07

**Published:** 2022-07-19

**Authors:** Casey Theriot, Rajani Thanissery, Sarah O'Flaherty, Rodolphe Barrangou

**Affiliations:** ^1^Department of Population Health and Pathobiology, College of Veterinary Medicine, North Carolina State University, Raleigh, NC 27695, USA.; ^2^Department of Food, Bioprocessing, and Nutrition Sciences, North Carolina State University, Raleigh, NC 27695, USA.

**Keywords:** Probiotics, antibiotics, colonization, microbiota

## Abstract

**Background: **The ability of probiotic strains to provide health benefits to the host partially hinges on the survival of gastrointestinal passage and temporary colonization of the digestive tract. This study aims to investigate the colonization profile of individual probiotic strains comprising the commercial product VSL#3^®^ and determine their impact on the host intestinal microbiota.

**Methods: **Using a cefoperazone-treated mouse model of antibiotic treatment, we investigated the impact of oral gavage with ~10^8^ CFU commercial VSL#3^®^ product on the intestinal microbiota using 16S-based amplicon sequencing over 7 days.

**Results: **Results showed that probiotic strains in the formulation were detected in treated murine fecal samples, with early colonization by *Streptococcus thermophilus *and* Lactiplantibacillus plantarum *subsp.* plantarum*, and late colonization by *Lacticaseibacillus paracasei *subsp.* paracasei*,* Bifidobacterium breve *and* Bifidobacterium animalis *subsp. *lactis. *Overall, VSL#3^®^ consumption is associated with increased alpha diversity in the cecal microbial community, which is important in the context of antibiotic consumption. Probiotic supplementation resulted in an expansion of Proteobacteria, Bacteroidetes, and Actinobacteria, especially Bifidobacteriaceae and Lachnospiraceae, which are associated with *Clostridioides difficile *resistance in the murine gut.

**Conclusion: **This study illustrates the need for determining the ability of probiotics to colonize the host and impact the gut microbiota, and suggests that multiple doses may be warranted for extended transient colonization. In addition, follow-up studies should determine whether VSL#3^®^ can provide resistance against *C. difficile *colonization and disease in a mouse model.

## INTRODUCTION

Over the past two decades, numerous studies have established intestinal microbial communities as important drivers of human health and disease^[1,2]^. DNA sequencing technologies providing high-throughput insights into the genetic content of complex samples, in combination with bioinformatic tools that enable interpretation of the phylogenetic groups comprising mixed populations have facilitated the analysis of complex microbial community composition^[3]^. Besides the understanding of human gut microbiota composition and function, there has been much interest in manipulation of the intestinal microbiota, notably through fecal microbiota transplantation (FMT), to drive the engraftment of healthy donor strains into diseased recipients^[4-6]^. The ability to detect and track strains being delivered to patients and consumers is important to determine their ability to transiently colonize or even durably engraft into recipients^[7]^, sometimes for multiple years^[4,8]^. Collectively, the microbiome literature has established that thousands of strains representing hundreds of bacterial species comprise the human gut microbiota^[4,9,10]^, though there is much variability in our understanding of their genetic composition and phenotypic functions, as well as our ability to culture them in the lab, let alone grow them at industrial scale. Besides manipulation of the human gut microbiota using additive approaches, there is likewise extensive literature related to the broad use of antibiotics to eradicate the microbial agents responsible for infectious disease. Several studies have shown that the broad range of antibiotics can have a negative impact on the composition of the gut microbiota, and there is much interest in developing means to reconstruct the gut microbiota post antibiotic consumption^[11,12]^. Altogether, the interplay between antibiotics, probiotics, indigenous gut bacteria, and pathogenic bacteria responsible for infectious disease is complex, dynamic, and hypervariable, but there are many efforts underway aiming at developing tools enabling the determination and manipulation of the human gut microbiome. 

Nevertheless, our overall understanding of high-resolution, strain-specific monitoring of microbiome dynamics in clinical research is still elusive, and recent advances in transcending operational taxonomic units (OTUs) illustrate the need for deciphering microbiota composition at the strain level to eventually account for function and not just composition of microbiomes^[13]^. To advance our understanding of gut health and disease, we must assess the impact of antibiotics on the gut microbiota and determine how this complex community can be reconstructed by the delivery of mixed bacterial communities that reconstitute a healthy and diverse microbiota. With studies showing variable results on the benefits and caveats of various probiotic formulations on the gut microbiota composition^[11,12]^, it is important to determine the extent to which orally-delivered probiotics can alter the gut microbiota post antibiotic treatment. Here, we use an established animal model of antibiotic-treated mice^[14]^ to determine the colonization dynamics of bacterial strains formulated in a commercial product following oral consumption over time^[15,16]^. We selected VSL#3^®^ as a broadly-studied commercial formulation with an established safety profile^[17]^, composition^[18]^, and documented benefits, notably regarding intestinal health^[19-23]^. Specifically, we determined the ability of the individual strains contained in a commercial sample of VSL#3^®^, consisting of a mixture of eight different bacterial strains, to colonize the intestinal tract of mice after treatment with antibiotic cefoperazone, and assessed the impact on the host microbiota.

## METHODS

### Animals and housing

C57BL/6J mice purchased from Jackson Laboratories (Bar Harbor, ME) were used for the experimental infections. Mice were housed with autoclaved food, bedding, and water. Cage changes were performed weekly in a laminar flow hood. Mice had a 12 h cycle of light and darkness. Mice were housed in a room with a temperature of 70 °F and 35% humidity. Animal experiments were conducted in the Laboratory Animal Facilities located on the NCSU College of Veterinary Medicine (CVM) campus. Animal studies were approved by NC State’s Institutional Animal Care and Use Committee (IACUC). The animal facilities are equipped with a full-time animal care staff coordinated by the Laboratory Animal Resources (LAR) division at NCSU. The NCSU CVM is accredited by the Association for the Assessment and Accreditation of Laboratory Animal Care International (AAALAC). Trained animal handlers in the facility fed and assessed the health status of animals daily. Those assessed as moribund were humanely euthanized by CO_2_ asphyxiation followed by secondary measures.

### The mouse model for probiotic colonization

**Figure 1:**
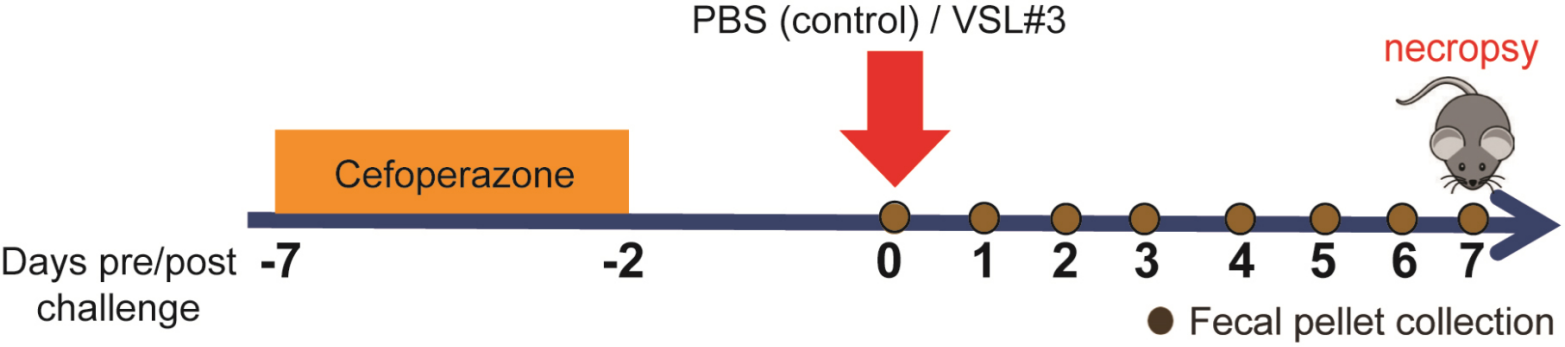
Antibiotic-treated mouse model treated with and without probiotic VSL#3^®^. Mouse model in which mice (*n* = 16) were randomly assigned to two groups. Antibiotic-treated mice were given cefoperazone in drinking water for 5 days and then a 2-day washout. Each group was challenged orally with VSL#3^®^ or PBS on day 0 and monitored post challenge for 7 days. Necropsy of mice was done on day 7 post challenge (*n* = 8 mice per treatment group, 4 males and 4 females). PBS: Phosphate-buffered saline.

The mouse model consisted of 5-week-old C57BL/6J mice (*n* = 16, 8 males and 8 females) randomly assigned into two groups [Figure 1]. Both groups consisted of mice that were given cefoperazone (0.5 mg/mL) in drinking water *ad libitum* for 5 days, followed by a 2-day washout with regular drinking water. On day 0, all mice were challenged via oral gavage with 100 µL of ~10^9^ CFU/mL VSL#3^®^, or 100 µL of phosphate-buffered saline (PBS) and monitored for 7 days. Fecal pellets were collected throughout the experiment and necropsy was performed on day 7. One VSL#3^®^ capsule was resuspended in 1.3 mL of PBS, and bacterial enumeration was done on MRS medium overnight at 37 °C in an anaerobic chamber. Approximately 10^9^ CFU/mL bacterial colonies were enumerated. This represented total bacteria counted and not individual bacterial strains. VSL#3^®^ is a high-concentration multi-strain probiotic mix containing one strain of *Streptococcus thermophilus* BT01, three strains of bifidobacteria (*B. breve* BB02; *B. animalis* subsp. *lactis* BL03, previously identified as *B. longum* BL03; and *B. animalis* subsp. *lactis* BI04, previously identified as *B. infantis* BI04), and four strains of lactobacilli (*L. acidophilus* BA05, *L. plantarum* BP06, *L. paracasei* BP07, and *L. helveticus* BD08, previously identified as *L. delbrueckii* subsp. *bulgaricus* BD08). VSL#3^®^ product used in this study were purchased at a local store (Lot n° 806084)

### Microbiota analysis

Fecal pellets and cecal tissue collected at necropsy were subjected to community 16S rRNA gene sequencing to determine overall microbiota composition. Microbial DNA was extracted from the fecal and cecal tissues using the MagAttract Power Microbiome kit. Analysis of the V4 region of the 16S rRNA gene was done in the statistical programming environment R using the package DADA2.44 Version 1.8 of the DADA2 tutorial workflow (https://benjjneb.github.io/dada2/tutorial.html)^[24,25]^. Sequencing analysis resulted in a final read depth ranging from 12,109-70,198 reads per sample, with an average of 38,723 reads and a median of 38,709 reads per sample. All statistical analyses of the microbiota profiles were performed in R, using packages as described below. The phyloseq and vegan packages were used to obtain diversity indices and ordination plots^[26]^. Associations within challenge days and between treatments with alpha diversity were measured by Kruskal-Wallis nonparametric statistical test for fecal samples, and Wilcoxon test for cecal samples. Associations with Bray-Curtis beta diversity were done by PERMANOVA using the adonis2 function from the vegan package. Differential-abundance analysis was performed using the ALDEx2 package^[27]^ and visualized with the ggplot2 package. The sequences from the V4 region of the 16S rRNA genes were classified into amplicon sequence variants (ASVs) and blasted against the V4 region of each bacterial strain present in the VSL#3^®^. All strains listed in the paper matched 100% sequence identity.

### PCR to detect probiotic strain

#### Lactobacillus acidophilus

PCR with primer pair; Laci_ABC_F (5’ AAA CTG CAA TTT AAG ATT ATG AGT TTC 3’) and Laci_ABC_R (5’ GGT ACC GTC TTG ATT ATT AGT GTA 3’) was performed to amplify a 610 bp* L. acidophilus*-specific amplicon from DNA extracted from fecal samples as detailed above. The primers used were synthesized by Integrated DNA Technologies (Coralville, IA, USA). The reaction products were separated on an agarose (VWR, PA, USA) gel and detected by ethidium bromide staining. Genomic DNA was used as a positive control and prepared by growing *L. acidophilus* overnight in MRS media at 37 °C. Subsequently, DNA was extracted from the overnight cultures using a DNeasy PowerLyzer microbial kit (Qiagen Valencia, CA, USA) as per the manufacturer’s instructions. Finally, DNA Sanger sequencing was performed to determine the nucleotide sequence of the amplicon (Genewiz, NC, USA) prior to BLAST analysis against the NCBI database (NCBI, MD, USA).

## RESULTS

### Colonization dynamics after oral inoculation of probiotics and associated changes in the gut microbiota in antibiotic-treated mice

**Figure 2:**
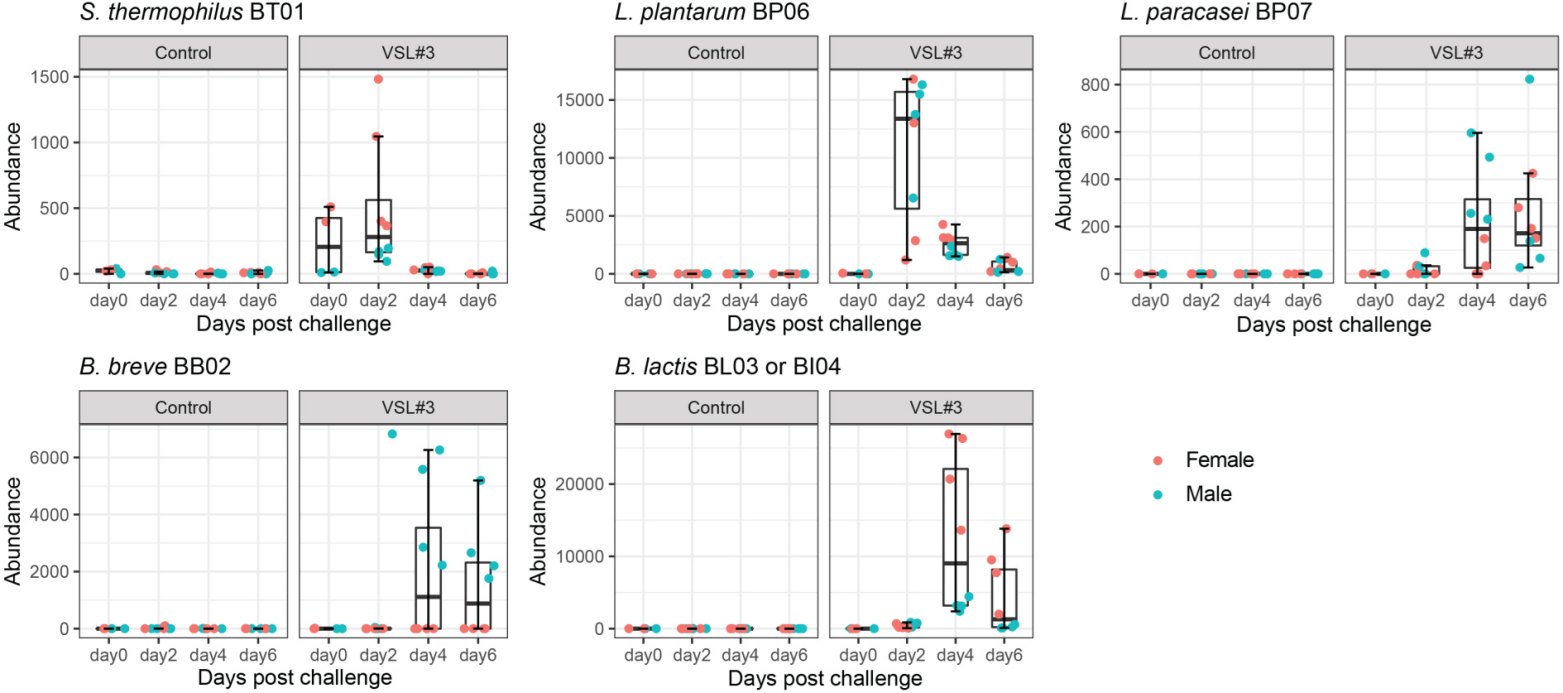
Fecal abundance of individual probiotic strains over time in antibiotic-treated mice. Fecal samples were collected on days 0, 2, 4, and 6 post challenge for microbiota analysis. The total read count of ASVs or each bacterial strain was determined by 16S rRNA gene sequencing analysis (*n* = 4-8 mice per treatment group). ASVs: Amplicon sequence variants.

The colonization dynamics of the eight probiotic strains in the feces over time (days 0, 2, 4, and 6 post challenge), and in ceca on day 7 post challenge were determined using 16S rRNA gene sequencing [Figure 2]. ASVs obtained from the resulting DADA2 sequence reads were used to identify probiotic strains. ASVs have a single-nucleotide resolution, which can allow for species-level classification by exact matching of V4 amplicon sequences. The DADA2 genus classification algorithm identified 5 ASVs belonging to the genus *Streptococcus*, 16 ASVs belonging to the genus *Lactobacillus*, and 4 ASVs belonging to the genus *Bifidobacterium. *From these sequences, the following exact matches from the reference genome for the probiotic strains were only identified in mice challenged with the probiotic compared to the PBS control. ASV 88 for *S. thermophilus* BT01, ASV 9 for *L. plantarum* BP06, ASV 64 for *L. paracasei *BP07, ASV 614 for *L. acidophilus* BA05, ASV 12 for *B. breve *BB02, and ASV 11 for *B. lactis. *ASV 11 did not distinguish between the two *B. lactis* strains, BL03 and Bl04. 

Abundance, as determined by 16S rRNA sequencing, revealed that six of the eight strains were detected from the fecal samples of the probiotic treated groups, and none were identified from the control group. *L. acidophilus* BA05 and *L. helveticus* BD08 were not detected in the 16S data, but BA05 DNA was recovered from several fecal samples using targeted PCR [Supplementary Figure 1]. Two types of colonization dynamics were observed. Early colonizing strains (*S. thermophilus* BT01 and *L. plantarum* BP06) showed higher abundance on day 2 post challenge in the probiotic treatment, which declined at later time points. A later colonization dynamic for *L. paracasei *BP07, *B. breve*, and *B. lactis* took up to 4 days to show detectable levels. 

**Figure 3:**
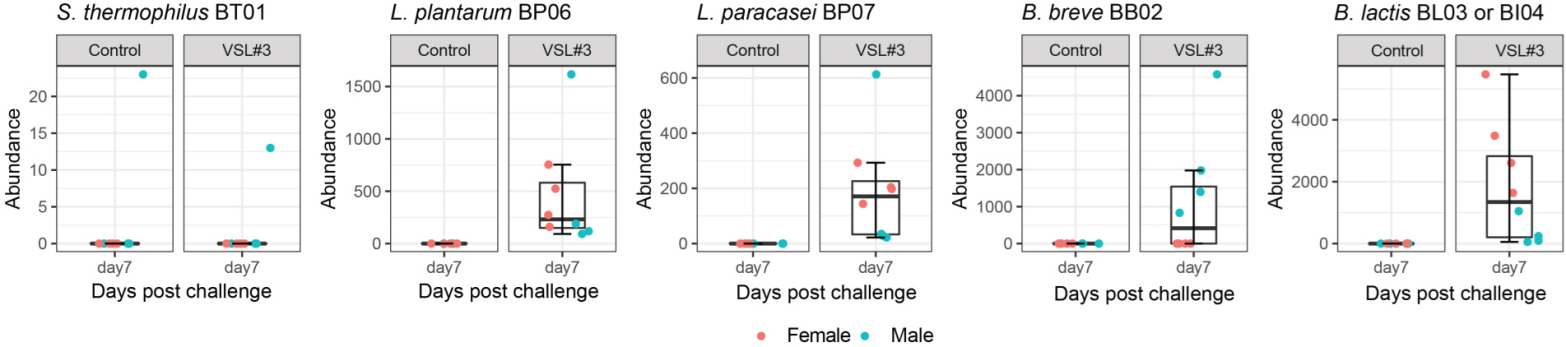
Cecal abundance of individual probiotic strains in antibiotic-treated mice. Cecal samples were collected on day 7 post challenge at necropsy for microbiota analysis. The total read count of ASVs or each bacterial strain was determined by 16S rRNA gene sequencing analysis (*n* = 4-8 mice per treatment group). ASVs: Amplicon sequence variants.

In the ceca of the antibiotic-treated mice, all probiotic strains except *L. helveticus* BD08 and *L. acidophilus* BA05 were recovered [Figure 3]. A similar trend was noticed where a higher abundance of *B. lactis *was found in the VSL#3^®^ group. *S. thermophilus* BT01 was only detected in a few samples, consistent with the aforementioned early colonizing dynamic which became reduced or undetectable by day 7 post challenge. 

Six of the eight probiotic strains from VSL#3^®^ samples administered as a single dose to mice were detectable in the antibiotic-treated mouse gut. One additional strain was detected using targeted PCR. Major differences in the gut microbiota were seen in the antibiotic-treated mice between the control and VSL#3^®^ treatments in feces over time and in the ceca of mice at day 7. Differential abundance data suggests that the probiotic strains are driving the changes in the microbial community structures in the VSL#3^®^ treatment. Colonization dynamics were altered by the product, with key drivers primarily consisting of Lachnoclostridium and Erysipelotrichaceae. The probiotic strains did not colonize the gut of wild-type mice, and no major changes were seen in the gut microbiota for this group.

### VSL#3^®^ treatment is associated with increased alpha diversity in the cecal microbial community

**Figure 4:**
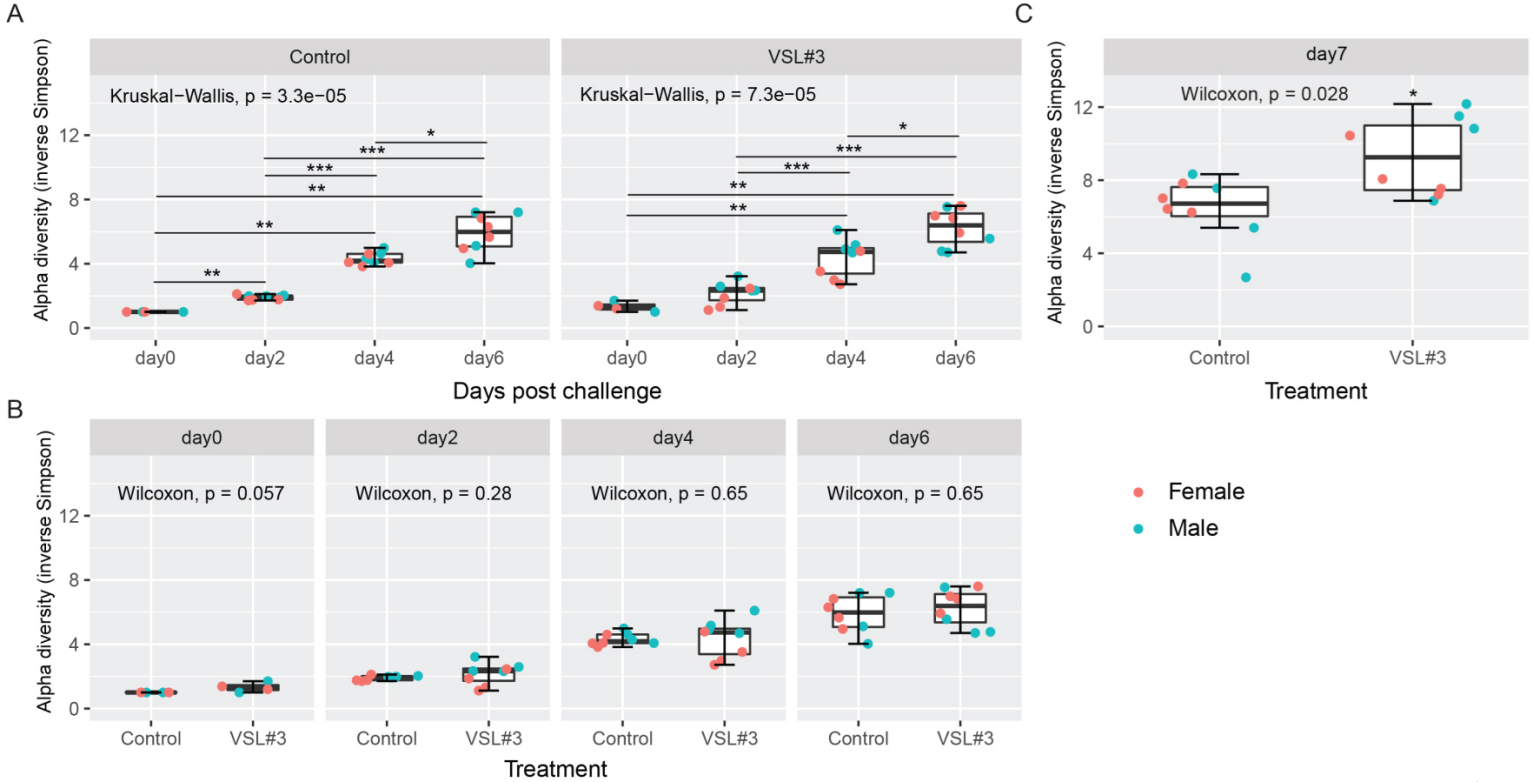
Probiotic treatment with VSL#3^®^ increases alpha diversity of the cecal microbiota on day 7 post challenge. Alpha diversity in the fecal microbiota was measured (A) over time between treatment groups and (B) between treatment groups by day. (C) Alpha diversity in the cecal microbiota compared for differences between treatments on day 7. Kruskal-Wallis one-way analysis of variance (ANOVA) followed by Dunn’s multiple comparison post hoc test was used in A (*P ≤ 0.05; **P ≤ 0.01, ***P ≤ 0.001). Wilcoxon rank sum test was used in B and C (***P ≤ 0.001). Error bars represent the standard deviation from the mean (*n* = 4-8 mice per treatment group).

Next, we assessed the variation in community profile with single-dose probiotic inoculation over time. The alpha diversity of the fecal and cecal microbiota was assessed using the inverse Simpson index, which incorporates both “richness” (the number of different bacterial species per sample) and “evenness” (the relative abundances of the different species making up the samples) of the sample. Alpha diversity was measured on days 0, 2, 4, and 6 post challenge for fecal samples, and on day 7 post challenge for cecal samples. The distributions of the inverse Simpson index at ASV levels are presented in Figure 4. For fecal samples, comparisons were made between days for each treatment [Figure 4A], as well as between treatment cohorts (Control *vs.* VSL#3^®^) within days [Figure 4B]. The diversity of microbial community is known to reduce significantly with antibiotic treatment and presents an increasing recovery tendency over time after the withdrawal of antibiotics. Higher diversity is often linked to a healthy state, and a decrease in diversity is associated with susceptibility to *C. difficile* infection after antibiotic treatment in a mouse model. As expected, an increasing alpha diversity over time was noticed in all groups. By day 7 post challenge, the VSL#3^®^ group showed the highest diversity within the ceca, indicating a positive effect on microbial recovery within the cecal community [Figure 4C].

### VSL#3^®^ treatment resulted in a distinct fecal community structure compared to the control

**Figure 5:**
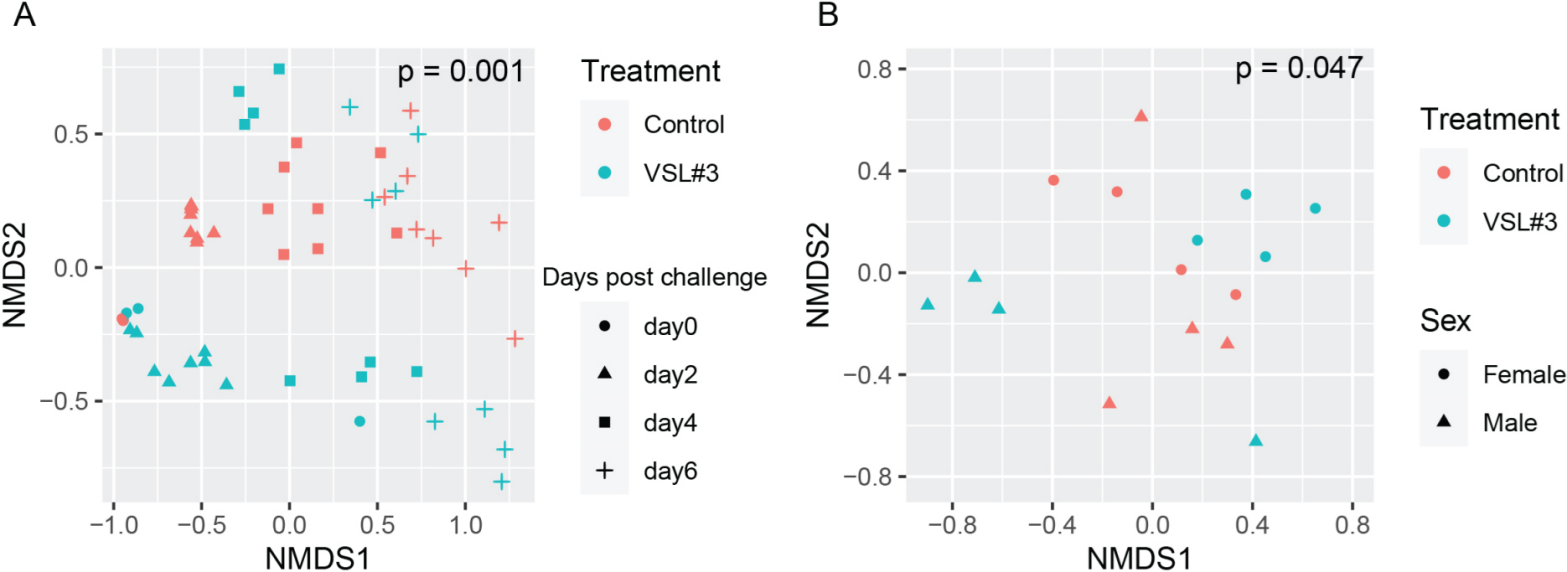
Beta-diversity in fecal and cecal microbiota measured over time. Using unsupervised clustering, NMDS illustrates the dissimilarity indices via Bray-Curtis distances between the bacterial communities from feces on days 0, 2, 4, and 6 post challenge (A) and from ceca on day 7 post challenge (B) (*n* = 4-8 mice per treatment group). NMDS: Non-metric multidimensional scaling.

To further elucidate factors related to the differences and similarities between fecal microbial community structures (β diversity), we performed non-metric multidimensional scaling (NMDS) based on Bray-Curtis dissimilarities between samples at the ASV level. A statistically significant shift in the fecal microbial community structures over time (days 0, 2, 4, and 6 post challenge) was noticed based on treatment groups [Figure 5A], and in the cecal microbial community on day 7 post challenge in Figure 5B.

**Figure 6:**
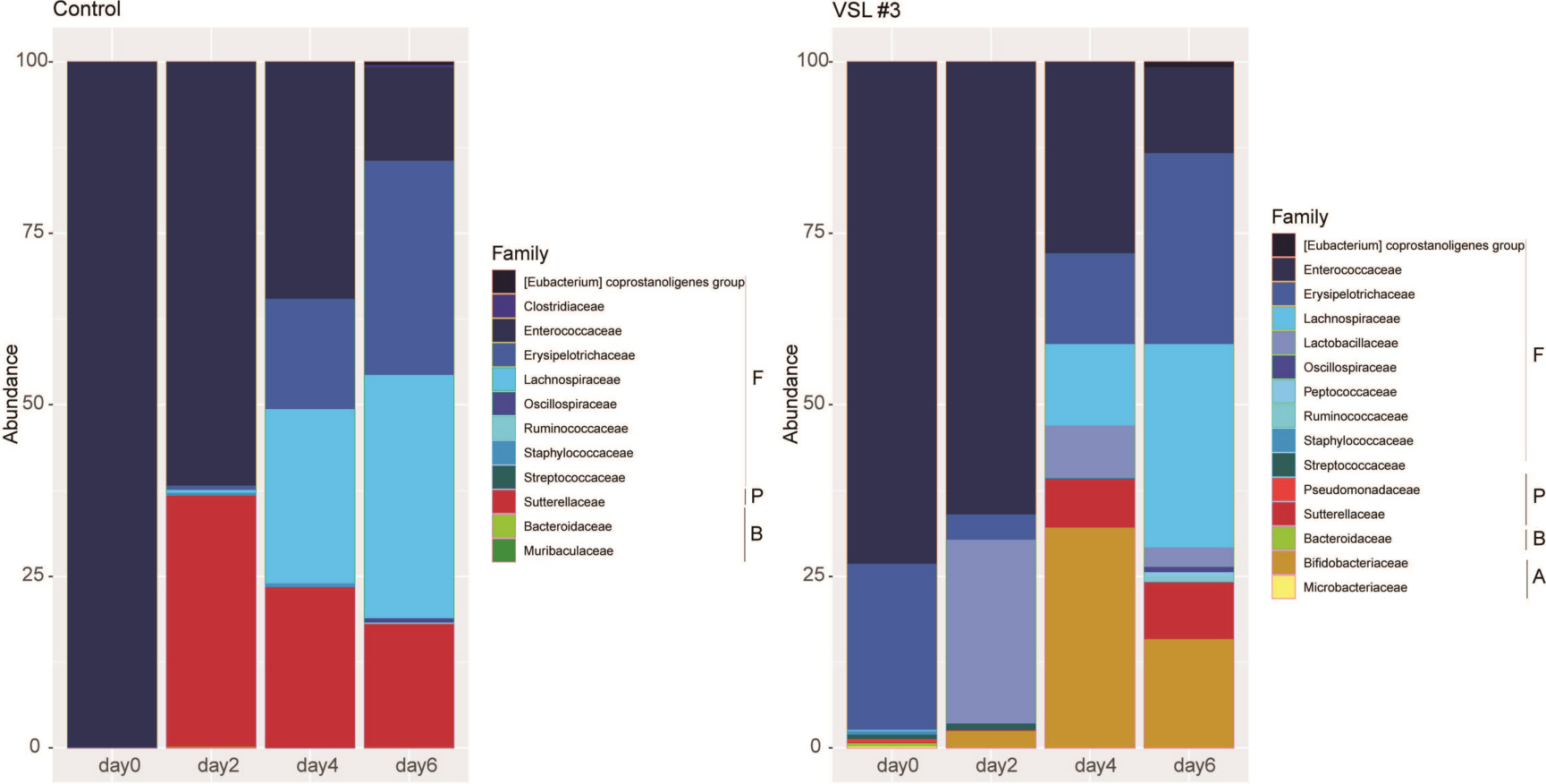
Impact of VSL#3^®^ on fecal bacterial community membership on days 0, 2, 4, 6 post challenge. Bar plots depict the mean percent abundances of the top bacterial families sorted by phylum where F - Firmicutes, P - Proteobacteria, B - Bacteroidetes, and A - Actinobacteria (*n *= 4-8 mice per treatment group).

We next investigated the differences in fecal bacterial community composition over time (days 0, 2, 4, and 6 post challenge) for the control and VSL#3^®^-treated groups [Figure 6]. Antibiotics are known to significantly reduce the diversity and alter the composition of the gut microbiota. This was reflected on day 0 (2 days post antibiotic cessation) by predominant representation by a single phylum Firmicutes in all groups.

**Figure 7:**
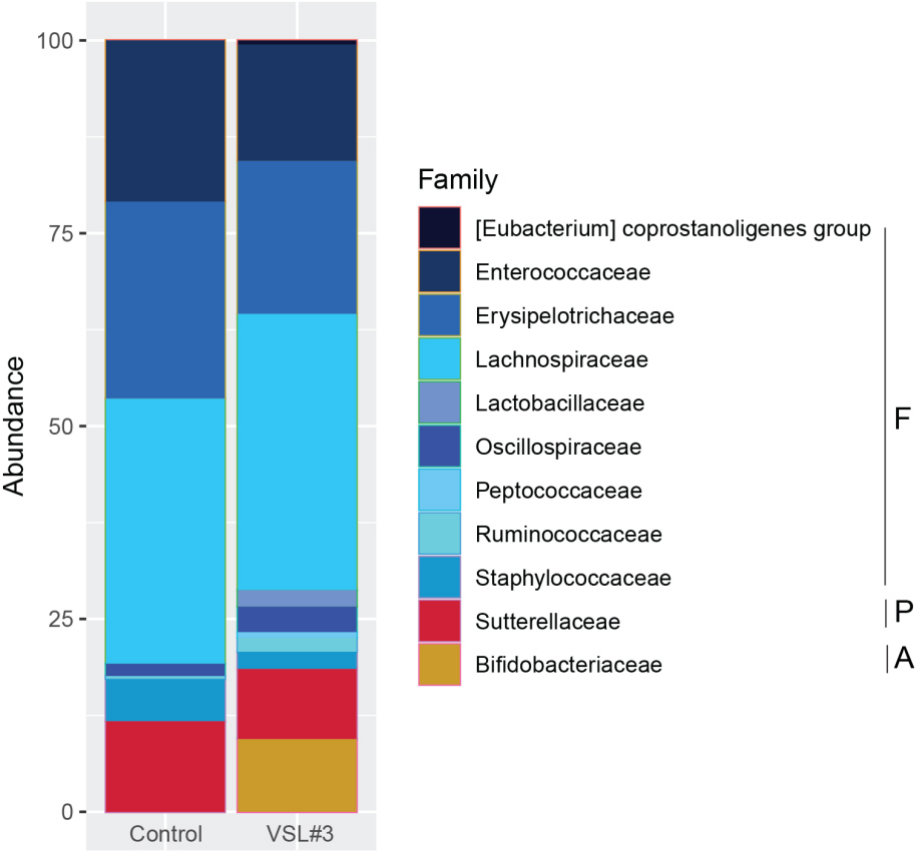
Impact of VSL#3^®^ on cecal bacterial community membership on days 7 post challenge. Bar plots depict the mean percent abundances of the top bacterial families sorted by phylum where F - Firmicutes, P - Proteobacteria, B - Bacteroidetes, and A - Actinobacteria (*n* = 4-8 mice per treatment group).

Over time, there was a slow return of several members of the phylum Firmicutes, Proteobacteria and Bacteroidetes in the feces of the control group that was administered PBS. Probiotic supplementation resulted in the expansion of the members of the phylum Proteobacteria, Bacteroidetes, and Actinobacteria. Supplementation with VSL#3^®^ resulted in the expansion of bacteria from the families Lachnospiraceae and Bifidobacteriaceae in the feces and the cecum on day 7 post challenge [Figures 6 and 7]. Members of the family Bacteroidaceae and Lachnospiraceae are associated with *C. difficile* resistance in the murine gut. The expansion of Bifidobacteriaceae could be from the three members of these taxa present in the administered probiotic product.

### Probiotic strains are driving the compositional changes in the VSL#3^®^ challenged mice

**Figure 8:**
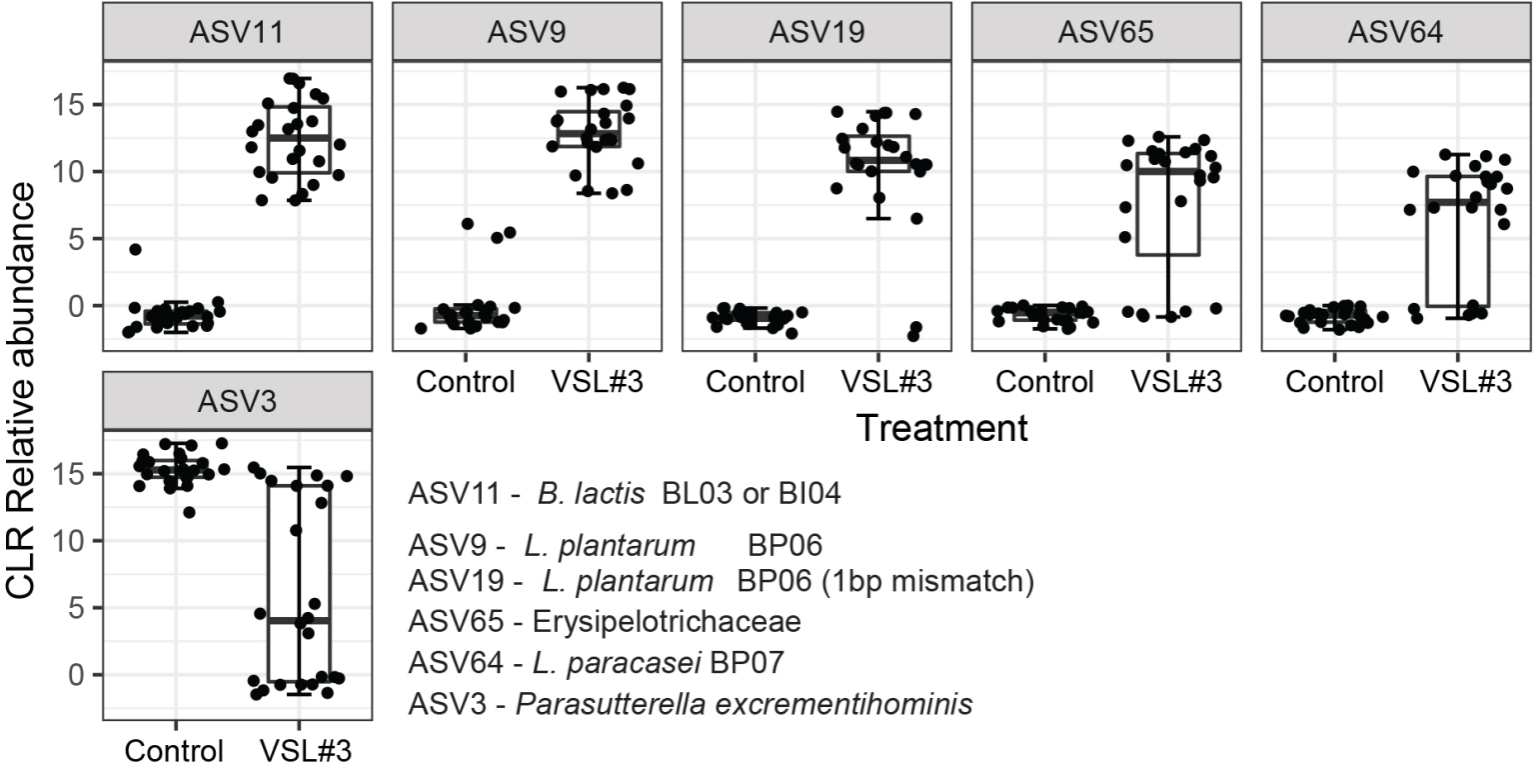
Fecal community composition differences between groups using a CLR transform of the ASV abundances. The presented ASVs are the largest positive and negative effect size that was differentially abundant with q < 0.1 in a MA effect plot. Points indicate the mean CLR value for each sample. CLR: Centered-log-ratio; ASVs: amplicon sequence variants.

Compositional difference in the fecal and cecal community with probiotic supplementation was evident from the relative abundance charts; therefore, we proceeded to determine the ASVs driving this variation. We performed a differential-abundance analysis between the control and VSL#3^®^ group with the ALDEx2 R package. This was performed for the combined time points of days 0, 2, 4, and 6 post challenge for the fecal samples and day 7 post challenge for the cecal samples. For each ASV, ALDEx2 reports an effect size estimating the difference in the taxon’s centered-log-ratio (CLR, a measure of relative abundance) between groups divided by the difference within groups. Relative abundances of the top positive and negatively associated ASVs are presented in Figure 8. 

**Figure 9:**
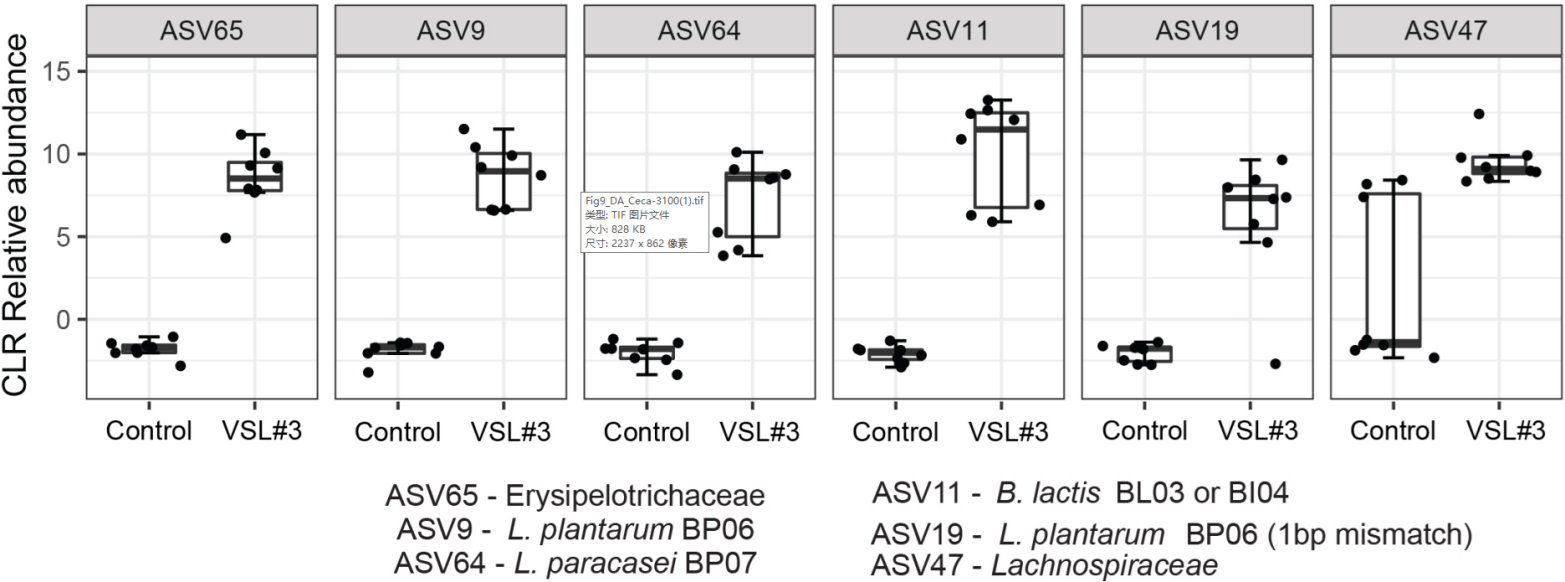
Cecal community composition differences between groups using a CLR transform of the ASV abundances. The presented ASVs are the largest positive and negative effect size that was differentially abundant with q < 0.1 in a MA effect plot. Points indicate the mean CLR value for each sample. CLR: Centered-log-ratio; ASVs: amplicon sequence variants.

The bar plots showing a striking increase in abundance with VSL#3^®^ treatment [Figure 8] were *B. lactis *Bl04 and BL03, *L. plantarum* BP06, *L. plantarum* BP06 (1 base pair mismatch), Erysipelotrichaceae, and - *L. paracasei* BP07. This indicates that the changes in community structures were mostly driven by the strains present in the probiotic mixture. A similar trend was noticed in the ceca of the VSL#3^®^ group, where an additional ASV belonging to the taxa Lachnospiraceae (ASV47) was also found to be positively correlated [Figure 9].

## DISCUSSION

In order to determine the impact of orally-consumed mixed microbial communities in commercial products, it is important to assess both the ability of these strains to survive intestinal passage and transiently colonize the host gastrointestinal tract, as well as ascertain their impact on overall community composition. Using high-level community profiling based on 16S sequencing, we show that of the eight individual strains present in the original product, most strains are detected post oral consumption in fecal samples with various time dynamics, encompassing early colonizers such as *S. thermophilus *and *L. plantarum*, as well as late colonizers including *L. paracasei*,* B. breve *and *B. lactis. *This is corroborated by the detection of these strains in the mouse cecum 7 days post oral consumption, revealing both survival and transient host colonization. The early and limited engraftment of lactobacilli and somewhat later and a relatively higher level of Bifidobacteria are consistent with long-term studies of FMT-based isolates in human clinical trials showing little engraftment by Lactobacillales and engraftment of Bifidobacteriales^[4]^. This is also consistent with the detection of *Lactobacillus*,* Bifidobacterium*, and *Streptococcus *in antibiotic-treated mouse fecal samples following consumption of probiotics^[11]^. It is important to note that differences may be inherent to the means of delivery, use of different antibiotics, and orally-consumed probiotic formulations would have benefits over colonoscope infusions of bacterial samples.

Importantly, the tracking of alpha diversity [Figure 4] and beta diversity [Figure 5] in the whole bacterial population revealed a beneficial increase of diversity over time, and more diversity in the VSL#3^®^ group than in the control [Figure 3], which is critical in the context of antibiotic treatment. Indeed, antibiotics typically reduce bacterial diversity dramatically and disrupt both composition and function of the microbiota in undesirable ways^[15,28]^. Besides the overall diversity, the actual microbial communities were distinct as determined by NMDS based on Bray-Curtis dissimilarities. Community composition analyses revealed that VSL#3^®^ most impacted the Proteobacteria, Bacteroidetes, and Actinobacteria Phyla [Figure 6], with a noteworthy expansion of Lachnospiraceae and Bifidobacteriaceae [Figure 7], presumably linked to the presence of *Bifidobacteria* in the product. These results are potentially clinically relevant given the association of these taxonomic families with *C. difficile* resistance in the murine gut^[2,15]^. Given the rise in awareness about the importance of *Bifidobacteria* in human health and disease, future studies should determine the functional attributes of these microbial community shifts. *Bifidobacteria*, in particular, could prove uniquely critical in the establishment of a healthy microbiota early in life, given their widespread occurrence and documented ability to impact infant health in the context of breastfeeding and milk digestion^[29]^. This is also critical given the impact of antibiotic exposure early in life and associated risks for subsequent development of disorders later in life^[30]^.

The variability in relative amounts and timing observed across the various strains in the commercial product is noteworthy, and the observed patterns are consistent with previous studies of probiotic consumption following antibiotic treatment^[11]^. In particular, this applies both to the limited ability of *L. acidophilus*, the intermediate ability of *L. plantarum *and* L. paracasei*, and the higher ability of *Bifidobacteria *to colonize mice^[11]^. Of course, differences may also be inherent to the specific strains that are used, and of course, the experimental setup implemented in terms of delivered probiotic amount, antibiotic type and dose, mouse genetic background and diet, and other factors. Determining whether and how quickly they could accelerate a return to a healthy composition is important, but these various factors have to be taken into account to disentangle the drivers of health and disease. There may be a need to balance addressing the disease-related issues inherent to pathogens and antibiotics on one side, and the health-related issues incumbent on a healthy and diverse gut microbiome on the other side. 

Since many FMT-focused studies have shown the potential to treat recurrent *C. difficile *infection, there is growing interest in manipulating the host microbiota to restore a healthy microbiome through the engraftment of health-promoting strains^[7]^. Given uncertainty and variability regarding the composition of FMT donor stool samples, there is rising interest in determining which individual bacterial strains have the potential for delivery to and engraftment into the host towards the development of defined cocktails of live biotherapeutic products as well-defined alternatives to FMT. Results shown here provide a potential basis to assess the potential of these strains for *C. difficile *therapeutics based on promoting diversity, with inherent advantages comprising established safety of human consumption and the ability to scale up manufacturing at large industrial scales. This study also highlights the need to develop and use strain-level genotyping methods to complement 16S-based sequences and ensure strain-level resolution using metagenomic approaches or strain-specific hypervariable sequences. Depth of sequencing should be considered to ensure proper coverage, and investigators may elect to corroborate molecular and sequence-based findings with microbiological analyses that encompass cell counts to quantify overall and relative amounts of bacteria of interest. Future studies should also consider determining the ability of individual strains of interest to both survive intestinal passage and their ability to transiently colonize the host.

In conclusion, the objective of the project was to test whether individual strains contained in commercial samples of VSL#3^®^ consisting of a mixture of eight different bacterial strains, are able to colonize the intestinal tract of mice. Overall, all probiotic strains except *L. helveticus *were able to colonize the antibiotic-treated mice gut (they were not detected in the control group), though strains did not colonize the gut of wild-type mice, and no major changes were seen in the gut microbiota for this group. Results suggest that multiple dosing of probiotic formulations may be warranted, given differences observed over time post treatment (from a single dose), to establish more stable and sustainable host colonization. An extended study could determine the long-term impact on microbiome composition and function reconstruction post antibiotic treatment, though it is unclear how translatable various timelines may be from mice to humans. Furthermore, given the widespread formulation of various *Streptococcus*,* Lactobacillus *and *Bifidobacterium *strains in human foods and dietary supplements, there is a need to assess strain-specific occurrence and diversity of members of the genera and species studied here in humans, especially since they can colonize the host for months. Noteworthy, the observed higher alpha diversity associated with the treatments is beneficial in the context of antibiotic treatment and also associated with reduced susceptibility to *C. difficile *infection. We suggest a follow-up study to test whether VSL#3^®^ can provide resistance against *C. difficile* infection in a mouse model, especially since the VSL#3^®^ group showed high diversity within the ceca.

## DECLARATIONS

### Availability of data and materials

All raw data is available upon request.

### Copyright

© The Author(s) 2022.
