# Supplementary material for: Probiotic colonization dynamics after oral consumption of VSL#3® by antibiotic-treated mice
Source: Microbiome Res Rep. 2022 Jul 19;1(4):21. doi: 10.20517/mrr.2022.07 (PMC10688816; doi:10.20517/mrr.2022.07)
Supplement: Supplementary file 1 [file mrr-1-4-21-SupplementaryMaterials.pdf]

A

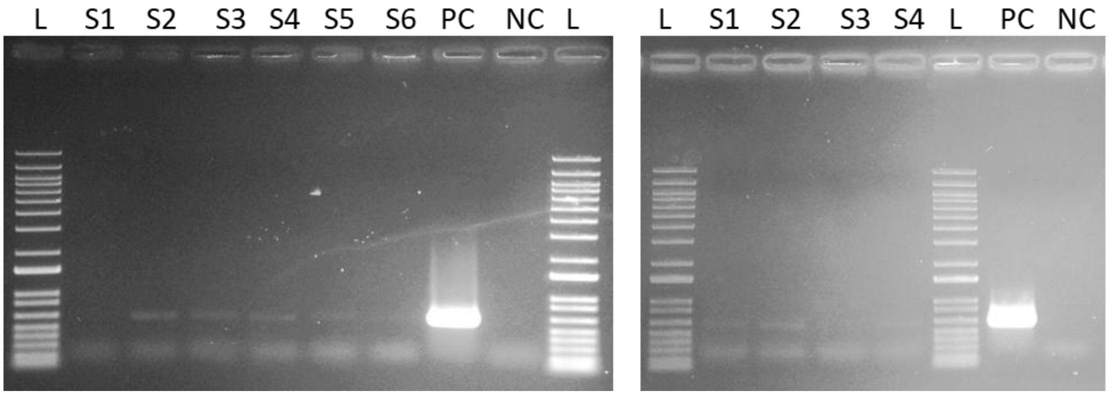

B

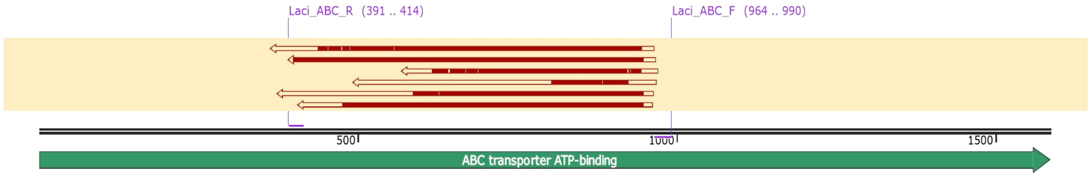

Supplementary Figure 1. Detection of *Lactobacillus acidophilus* using targeted PCR on DNA extracted from fecal samples from VSL#3 treated mice. (A) Detection of a 610 bp amplicon in the six samples (S2 to S6 in the gel to the left and S1 to S4 in the gel on the right). PC, positive control (*L. acidophilus* DNA). NC, negative control and L, 1 kb plus ladder (Thermo Fisher Scientific). Ten ng of DNA isolated from six fecal samples of VSL#3 treated mice was used in PCR reactions with primer pair Laci_ABC_F and Laci_ABC_R [Table 1]. Across two PCR reactions, the expected amplicon size (610 bp) was detected in all six samples. Although the PCR amplicon was weak, DNA Sanger sequencing was successfully performed on five of the six sample amplicons. (B) Using BLAST all five sequences were shown to align to *L. acidophilus* strains in the NCBI database. Alignments against the *L. acidophilus* ABC transporter gene confirmed the amplicon matched the expected *L. acidophilus* nucleotide sequence. Alignment of the DNA Sanger sequencing results are shown in order from top to bottom (S2 to S6 and PC). We expect that the amplicon in S1 also detected *L. acidophilus* but the DNA sequencing was not successful for this sample given the low amount and quality of extracted amplicon observed for all 6 samples.
